# The effect of a new direct Factor Xa inhibitor on human osteoblasts: an in-vitro study comparing the effect of rivaroxaban with enoxaparin

**DOI:** 10.1186/1471-2474-12-247

**Published:** 2011-10-28

**Authors:** Gandhi N Solayar, Pauline M Walsh, Kevin J Mulhall

**Affiliations:** 1 UCD Clinical Research Centre, UCD School of Medicine and Medical Sciences, Mater Misericordiae University Hospital, Dublin, Ireland; 2 Department of Orthopaedic Surgery, Mater Misericordiae University Hospital, Dublin 7, Ireland

## Abstract

**Background:**

Current treatments for the prevention of thromboembolism include heparin and low-molecular weight heparins (LMWHs). A number of studies have suggested that long term administration of these drugs may adversely affect osteoblasts and therefore, bone metabolism. Xarelto™ (Rivaroxaban) is a new anti-thrombotic drug for the prevention of venous thromboembolism in adult patients undergoing elective hip and knee replacement surgery. The aim of this *in vitro *study was to investigate the possible effects of rivaroxaban on osteoblast viability, function and gene expression compared to enoxaparin, a commonly used LMWH.

**Methods:**

Primary human osteoblast cultures were treated with varying concentrations of rivaroxaban (0.013, 0.13, 1.3 and 13 μg/ml) or enoxaparin (1, 10 and 100 μg/ml). The effect of each drug on osteoblast function was evaluated by measuring alkaline phosphatase activity. The MTS assay was used to assess the effect of drug treatments on cell proliferation. Changes in osteocalcin, Runx2 and BMP-2 messenger RNA (mRNA) expression following drug treatments were measured by real-time polymerase chain reaction (PCR).

**Results:**

Rivaroxaban and enoxaparin treatment did not adversely affect osteoblast viability. However, both drugs caused a significant reduction in osteoblast function, as measured by alkaline phosphatase activity. This reduction in osteoblast function was associated with a reduction in the mRNA expression of the bone marker, osteocalcin, the transcription factor, Runx2, and the osteogenic factor, BMP-2.

**Conclusions:**

These data show that rivaroxaban treatment may negatively affect bone through a reduction in osteoblast function.

## Background

Venous thromboembolism is a significant potential complication following orthopaedic surgery and an important cause of morbidity and mortality in adults. In the absence of thromboprophylaxis, the rate of deep vein thrombosis following major lower extremity orthopaedic surgery is between 40-60%, while the risk of developing fatal pulmonary embolism is between 1-2% [1,2]. Current treatments for the prevention of venous thromboembolism include heparin and low-molecular weight heparins (LMWHs). While heparin and LMWH therapy are effective measures for the prevention of thromboembolism, a number of studies have suggested that long term administration may negatively affect bone and some have associated their use with the risk of developing osteoporosis [3,4]. Although heparin-induced osteoporosis is a rare adverse effect, its incidence is thought to be in the range of 2-5% [5]. Studies have shown that prolonged unfractionated heparin treatment is associated with bone loss and an increased risk of fracture [6,7]. Studies have also shown that women who receive extended heparin therapy during pregnancy, have significant reduction of bone density in their lumbar spines [8]. There is some evidence to suggest that LMWH may have a reduced incidence of osteopenia and osteoporosis when compared with unfractionated heparin therapy [3].

Rivaroxaban or Xarelto™ (Bayer Schering Pharma AG) is an anti-thrombotic drug that was granted marketing approval by the European Commission (Enterprise & Industry/Pharmaceuticals) in 2008 for the prevention of venous thromboembolism in adult patients undergoing elective hip and knee replacement surgery [9]. A direct Factor Xa inhibitor, this drug represents an attractive alternative to heparin and LMWH for the purposes of prophylactic anti-coagulation as it is administered orally and thus removes the need for daily injections.

Rivaroxaban therapy is recommended for 5 weeks post hip replacement surgery and 2 weeks post knee replacement surgery [10]. In light of the fact that heparin and LMWHs have been associated with adverse effects on bone and a risk of developing osteoporosis, the aim of this study was to investigate the effects of rivaroxaban, compared with enoxaparin, on human osteoblasts *in vitro*. The effect of each drug was evaluated in terms of its effect on osteoblast viability, function and gene expression.

## Methods

### Osteoblast cell culture and treatment

A human osteoblast cell line (primary cells) was purchased from PromoCell (Heidelberg, Germany). Osteoblasts were isolated from bone biopsies (cancellous bone) obtained from the femoral condyles of the knee joint of a patient undergoing knee surgery (*N *= 1, 78 year old, Caucasian female). Osteoblast cells were characterised by the supplier as staining positive for alkaline phosphatase and osteocalcin. Cells were routinely cultured in Osteoblast Growth Media (C-27001; Promocell, Germany) at 37°C in a humidified atmosphere of 95% air and 5% CO_2_. Growth media contained 10% fetal calf serum, 125 μg/ml sodium phosphate and 50 μg/ml ascorbic acid. Cells were seeded into 6-well plates at a density of 1 × 10^5 ^cells (alkaline phosphatase assay, PCR analysis) or into 96-well plates at a density of 5 × 10^4 ^cells per well (cell viability assay). Cells were then cultured to approximately 80% confluence prior to treatment.

Osteoblast cells were cultured in the presence of enoxaparin (Clexane™, 100 IU/mg anti-Xa activity; Sanofi Aventis) at concentrations of 1, 10 and 100 μg/ml. Cells cultured in the presence of osteoblast growth medium alone served as experimental controls. Rivaroxaban (BAY 59-7939) was obtained directly from Bayer^® ^pharmaceuticals (Germany) in aliquots containing 10 mg of rivaroxaban salt. Rivaroxaban was dissolved in dimethyl sulphoxide (DMSO; Sigma, Ireland) as demonstrated in previous *in vitro *studies with this drug [11]. Final rivaroxaban concentrations were obtained by dilution in culture media and were as follows: 0.013, 0.13, 1.3 and 13 μg/ml, which included the mean *in vivo *therapeutic range (0.02 - 0.05 μg/ml) [12]. Cells cultured in media containing equivalent amounts of DMSO as treatments served as experimental controls for the rivaroxaban group. DMSO concentrations for each concentration of rivaroxaban treated cells were 0.182, 1.82, 18.2 and 182 mM, respectively. The need for two control groups was necessary as rivaroxaban required additional DMSO as a solvent while enoxaparin was completely soluble in osteoblast growth medium alone. During the one week treatment period, the culture medium was replaced every 72 hours with medium containing the test drug.

### Cell viability analysis

Cell viability was assessed using the CellTiter 96^® ^AQueous One Solution Cell Proliferation Assay (Promega, UK) as per manufacturer's instructions and previous literature [13]. It is a colorimetric assay and contains a tetrazolium compound that, when reduced, produces a colored formazan product that is soluble in cell culture medium and absorbs maximally at 490 nm. The compound is reduced as a result of the mitochondrial activity in the cells, and the amount of formazan that is produced is directly proportional to the number of viable cells Cells were seeded into 96-well plates at a density of 5 × 10^4 ^cells/well and cultured at 37°C in a humidified incubator containing 5% CO_2_. Once approximately 80% confluent, cells were treated for 72 hours with varying concentrations of enoxaparin or rivaroxaban and their respective controls. A 20 μl aliquot of the CellTitre 96^® ^Aqueous One Solution reagent was added to each culture well and after 4 hours the absorbance was recorded at 490 nm. The cell viability of treatment cultures was reported as a percentage of control cultures. All assays were carried out in triplicate.

### Assay for alkaline phosphatase activity

Alkaline phosphatase activity was measured as a marker of osteoblast function. Cells were seeded into 6-well plates at a density of 1 × 10^5 ^cells/well. Following 7 days of treatment with enoxaparin or rivaroxaban and their respective controls, cell lysates were collected by CelLytic M (Sigma, UK) and the extracted proteins frozen at -80°C. The alkaline phosphatase activity of cell lysates was determined using a *p-nitrophenylphosphate *(pNPP) based assay [14]. A 5 μl aliquot of cell lysate was incubated with 100 μl of p-NPP solution for 30 minutes at 37°C and the absorbance at 405 nm was recorded using a plate reader. Alkaline phosphatase activity was normalised to total protein, which was determined using the Bradford assay. The alkaline phosphatase activity of treatment cultures was expressed as a percentage of control cultures (n = 6; 6 independant cultures from a single donor cell source).

### RNA isolation and real-time PCR

Total cellular RNA was extracted from osteoblasts treated with enoxaparin or rivaroxaban and their respective controls using the TRI reagent (Sigma, Ireland), according to the manufacturer's instructions. Contaminating genomic DNA was removed from RNA samples using a DNA-*free*™ kit (Applied Biosystems, UK). RNA was reverse transcribed to complementary DNA (cDNA) using the Enhanced Avian Reverse Transcriptase kit (Sigma, Ireland). cDNA served as template for real-time PCR, which was conducted using the QIAGEN QuantiTect SYBR Green PCR kit (Qiagen, UK). Using gene specific primer pairs, the mRNA expression of the runt-related transcription factor 2 (Runx2), bone morphogenetic protein-2 (BMP-2) and Osteocalcin (OC) gene products were measured using absolute quantification and were reported as a function of crossing time (C_t_), the cycle number at which PCR amplification becomes linear. mRNA expression was normalised to control and glyceraldehyde-3-phosphate dehydrogenase (GAPDH) expression resulting in mean fold change values. Assays were carried out in triplicate (3 independent cDNA preparations extracted from 3 independent cultures) (Table 1 Table 2).

**Table 1:** Forward and reverse primers used in real-time PCR analysis

Gene	Forward	Reverse
Runx2	5'-TTACTTACACCCCGCCAGTC-3'	5'-TATGGAGTGCTGCTGGTCTG-3'
BMP-2	5'-TCAAGCCAAACACAAACAGC-3'	5'-AGCCACAATCCAGTCATTCC-3'
OC	5'-GACTGTGACGAGTTGGCTG -3'	5'-CTGGAGAGGAGCAGAACTGG-3'
GAPDH	5'-GAGTCAACGGATTTGGTCGT-3'	5'-TTGATTTTGGAGGGATCTCG-3'

**Table 2:** Average Ct values obtained for GAPDH during RT-PCR analysis

	Average Ct	
	Day 1	Day 7
Control	17.35	18.32
Enoxaparin 1 μg/ml	16.67	18.68
Enoxaparin 10 μg/ml	16.47	16.86
Enoxaparin 100 μg/ml	17.70	17.33
		
DMSO 0.182 mM	17.05	19.81
DMSO 1.82 mM	17.27	16.96
DMSO 18.2 mM	17.22	17.48
DMSO 182 mM	16.66	17.32
		
Rivaroxaban 0.013 μg/ml	16.07	17.68
Rivaroxaban 0.13 μg/ml	17.08	16.58
Rivaroxaban 1.3 μg/ml	16.72	15.32
Rivaroxaban 13 μg/ml	16.51	15.84

### Statistical Analysis

Data are given as a mean ± standard deviation. Data were analysed using Student's t-test to determine a significant difference between sample means. Differences were considered significant if *P *< 0.05.

## Results

### Effect of enoxaparin and rivaroxaban on osteoblast cell viability

**Figure 1:**
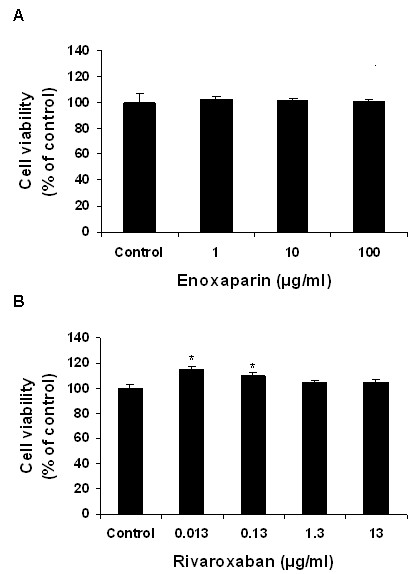
**Effect of enoxaparin and rivaroxaban on cell viability of human osteoblast cultures**. Human osteoblast cells were cultured in the presence of varying concentrations of (A) enoxaparin and (B) rivaroxaban for 72 hours after which cell viability was assessed using the MTS assay. Percent viable cells (y) are presented as a proportion of the viability of control cultures. Data are presented as the mean ± the standard deviation. *p < 0.05 from Student's t-test relative to the control treatment (n = 3).

From the results of the CellTiter 96^® ^AQueous One Solution Cell Proliferation Assay (Promega, UK), cells treated with enoxaparin were found to have a similar viability when compared to control cells (Figure 1). Rivaroxaban treatment did not have an adverse effect on osteoblast viability, however, cells treated with the lower concentrations of rivaroxaban (0.013 and 0.13 μg/ml) showed a significant increase in viability of 15% and 10%, respectively (Figure 1).

### Effect of enoxaparin and rivaroxaban on osteoblast function

**Figure 2:**
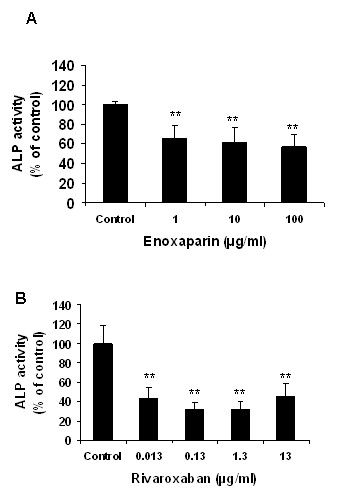
**Effect of enoxaparin and rivaroxaban on human osteoblast function**. Human osteoblast cultures were treated with varying concentrations of enoxaparin and rivaroxaban for 7 days and then analysed for alkaline phosphatase activity. The alkaline phosphatase activity of treatment cultures is expressed as a percentage of untreated controls. Data are presented as the mean ± the standard deviation. *p < 0.05; **p < 0.01 from Student's t-test relative to the control treatment (n = 6).

To determine the functional effect of enoxaparin and rivaroxaban treatment we measured the alkaline phosphatase activity after 7 days of treatment. Enoxaparin treatment induced a dose-dependent reduction in alkaline phosphatase activity, a well-established marker of osteoblast function (Figure 2). This reduction in alkaline phosphatase activity ranged from 35% +/-18% (p < 0.0001) to 50% +/- 19% (p < 0.0001). Rivaroxaban treatment resulted in a dramatic decrease in alkaline phosphatase activity at each of the concentrations tested when compared to control cultures (Figure 2). The lowest concentration of rivaroxaban caused a significant reduction in alkaline phosphatase activity (57% +/- 15%, p < 0.0001), with further reductions observed in the presence of higher rivaroxaban concentrations [0.13 μg/ml (69% +/- 10%, p < 0.0001) and [1.3 μg/ml (68% +/- 9%, p < 0.0001)]. When therapeutic levels of enoxaparin (1-10 μg/ml) and rivaroxaban (0.013-0.13 μg/ml) were compared, alkaline phosphatase activity was found to be significantly less in rivaroxaban-treated cultures at both the lower and higher therapeutic levels (enoxaparin 1 μg/ml versus rivaroxaban 0.013 μg/ml, p < 0.0005; enoxaparin 10 μg/ml versus rivaroxaban 0.13 μg/ml, p < 0.001).

### Effect of rivaroxaban and enoxaparin on osteocalcin expression

**Figure 3:**
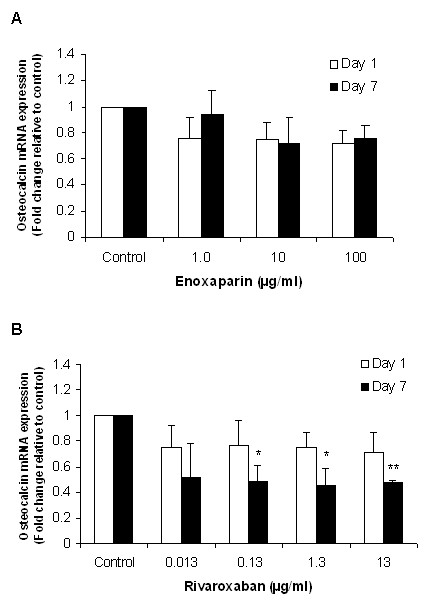
**(A&B) - Effect of enoxaparin and rivaroxaban on osteocalcin mRNA expression in human osteoblast cultures**. Following 1 and 7 days of treatment, osteoblast cultures were analysed for changes in osteocalcin mRNA expression using RT-PCR. Data are presented as mean fold change ± the standard deviation. *p < 0.05; **p < 0.01 from Student's t-test relative to the control treatment (n = 3).

To examine the effect of enoxaparin and rivaroxaban on osteocalcin, a marker of bone formation, we measured osteocalcin mRNA expression by real-time PCR following treatment with both drugs. Changes in gene expression were measured after 1 and 7 days in order to examine the immediate and long term effect of drug treatments. As shown in Figure 3 enoxaparin treatment led to a down-regulation of osteocalcin mRNA expression at both time-points, however, this reduction was not statistically significant. Rivaroxaban treatment resulted in a reduction in osteocalcin expression at all concentrations tested with a statistically significant reduction observed in the presence of higher rivaroxaban concentrations (0.13 - 13 μg/ml) after 7 days (Figure 3).

### Effect of enoxaparin and rivaroxaban treatment on Runx2 and BMP-2 expression

**Figure 4:**
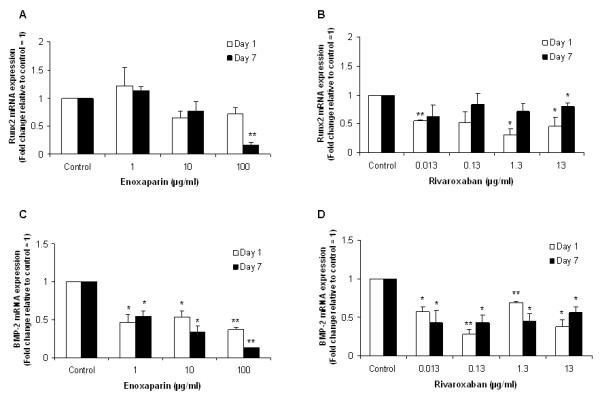
**The effect of enoxaparin and rivaroxaban concentration on Runx2 and BMP2 mRNA expression following 1 and 7 days of treatment**. Data are presented as mean fold change ± the standard deviation. *p < 0.05; **p < 0.01 from Student's t-test relative to the control treatment (n = 3).

We next determined the effect of both drugs on the major osteoblast transcription factor, Runx2, and the pro-osteogenic growth factor, BMP-2. A significant reduction in Runx2 mRNA expression was observed in cultures treated with the highest concentration of enoxaparin (100 μg/ml), and in cultures treated with a range of rivaroxaban concentrations, including the therapeutic levels (Figure 4 and 4). A significant reduction in BMP-2 mRNA expression was observed after 1 and 7 days of treatment in all enoxaparin and rivaroxaban treated cultures, as is illustrated in Figure 4 and 4.

## Discussion

Long term administration of heparin and LMWH has been associated with a negative effect on bone and an increased risk of developing osteoporosis [3,4]. Xarelto™ (Rivaroxaban) is a new anti-thrombotic drug that was recently licensed for the prevention of venous thromboembolism in adult patients undergoing elective hip and knee replacement surgery. At present, the effects of rivaroxaban on bone and osteoblasts are unknown. In this study, we investigated the effect of rivaroxaban on human osteoblasts in terms of its effect on viability, function and gene expression. Acute and chronic changes in gene expression were measured after 1 and 7 days treatment whereas the functional markers (alkaline phosphatase) were measured following 7 days of treatment. In addition, the effects induced by rivaroxaban were compared to those induced by enoxaparin, a commonly used LMWH. The principal finding of this study was that, rivaroxaban treatment leads to a reduction in osteoblast function, as measured by alkaline phosphate activity, and that this was associated with reduced expression of the bone marker, osteocalcin, the major osteoblast factor, Runx2, and the osteogenic factor, BMP-2.

Clinical trials comparing the efficacy and side effects of enoxaparin and rivaroxaban therapy on the outcome of thrombosis following joint replacement surgery favour the use of rivaroxaban for the prevention of thrombotic events post-arthroplasty (RECORD trials) [10]. This, coupled with the fact that rivaroxaban is available orally (eliminating the need for invasive administration), makes the use of rivaroxaban an attractive option for both orthopaedic surgeons and patients alike. Enoxaparin is generally administered for 5 to 7 days in the immediate post-operative period while the patient remains in hospital. In the case of rivaroxaban, therapy is recommended for 5 weeks post hip replacement surgery and 2 weeks post knee replacement surgery [10]. As long term administration of heparin and LMWH has been associated with reduced bone mineral density [8,15] and increased fracture rates in pregnant women [16,17], we investigated the effect of rivaroxaban on human osteoblasts.

In this study, neither enoxaparin nor rivaroxaban treatment caused a reduction in osteoblast cell viability indicating that both drugs do not show cytotoxic effects to osteoblasts studied. Our findings in relation to enoxaparin are in accordance with those of other studies, which have shown that enoxaparin does not have a cytotoxic effect on osteoblast viability [18]. Similarly, other low molecular weight heparins, such as fondaparinux, do not induce cytotoxic effects on osteoblast proliferation [19]. However, while rivaroxaban and enoxaparin treatment did not reduce osteoblast proliferation, both drugs caused a reduction of an established marker of osteoblast function: alkaline phosphatase. The bone-specific isoform of alkaline phosphatase is a tetrameric glycoprotein found on the surface of osteoblast cells and is believed to have a significant function in the mineralization of bone matrix [20]. Previous studies have shown that heparin and low-molecular-weight heparins, such as enoxaparin, exert a negative effect on alkaline phosphatase expression [21,22]. In the present study, both enoxaparin and rivaroxaban treatment caused a significant reduction in osteoblast alkaline phosphatase activity, with rivaroxaban causing a more negative effect. Osteocalcin is another commonly used marker of bone formation/turnover [23]. Osteocalcin expression was also reduced by enoxaparin and rivaroxaban treatment. This decrease was found to be statistically significant following treatment with rivaroxaban for 7 days indicating that prolonged rivaroxaban therapy may have a negative impact on osteoblast function.

Our findings of reduced alkaline phosphatase activity and osteocalcin expression in the absence of reduced cell viability suggested that enoxaparin and rivaroxaban treatment may negatively affect bone through a reduction in osteoblast function rather than a decrease in osteoblast proliferation. Therefore, to determine if rivaroxaban and enoxaparin influence osteoblast function through changes in osteoblast signalling, the expression of the transcription factor, Runx2, and the pro-osteogenic growth factor, BMP-2 were investigated. The exposure of human osteoblasts to enoxaparin and rivaroxaban resulted in a significant reduction of both Runx2 and BMP-2 expression in this study. Runx2 is the main transcription factor responsible for the development and maintenance of the osteoblast phenotype [24] and targeted disruption of this gene in mice leads to a complete lack of ossification [25]. The expression and activation of the Runx2 transcription factor is regulated by a number of bone-derived growth factors, including BMP-2 [26]. BMP2 has previously been shown to play a crucial role in bone formation and repair [27], and also in the regulation of osteoclastogenesis [28].

These findings suggest that enoxaparin and rivaroxaban may affect osteoblast function by reducing BMP-2 induced bone formation. Since the exposure of human osteoblasts to both drugs did not result in a cytotoxic affect in this study, a downregulation of BMP-2 signalling may explain the reduction in osteoblast functionality. Runx2 regulates the expression of several major extracellular matrix genes expressed by osteoblasts including alkaline phosphatase and osteocalcin [29,30]. Thus, a reduction in BMP-2 and Runx2 signalling, could lead to a reduction in alkaline phosphatase activity and osteocalcin expression, and ultimately lead to an impairment of osteoblast function. Studies have demonstrated that heparin can inhibit BMP-2 osteogenic activity by binding to both the BMP-2 and BMP receptor, and this effect has been associated with reduced Runx2, osteocalcin and alkaline phosphatase expression [31]. However, this type of BMP-2 mediated repression has not been reported in relation to enoxaparin or rivaroxaban.

Bone metabolism is a continuous remodelling process involving both bone formation by osteoblasts and bone resorption by osteoclasts. Heparin has been found to negatively affect bone formation by promoting the activation of osteoclasts and decreasing bone volume in rats, by inducing bone resorption in rat osteoclasts *in vitro *[32,33] and enhancing osteoclastic bone resorption through an inhibition of osteoprotegerin activity [34]. While the effect of enoxaparin and rivaroxaban on osteoclast function was not investigated as part of this study, our study confirmed that both thromboprophylactic agents negatively affect osteoblast function. Such findings indicate that long term therapy with enoxaparin or rivaroxaban could potentially translate into clinical effects on bone homeostasis.

## Conclusions

In conclusion, rivaroxaban and enoxaparin treatment led to a reduction in alkaline phosphatase activity and a reduction in BMP-2, osteocalcin and Runx2 mRNA expression, indicating that treatment with both drugs leads to a general negative effect on osteoblast activity. Due to the increased duration of therapy (as compared to enoxaparin), rivaroxaban could potentially be more detrimental than enoxaparin in terms of its effects on osteoblast function. While the findings of this study indicate that rivaroxaban negatively affects osteoblast function, further clinical investigations are required to elucidate whether these effects of rivaroxaban therapy translate into demonstrable effects on bone homeostasis *in vivo*. Given that these agents are typically promoted for use following hip and knee arthroplasty, and that many modern hip and knee implants are 'uncemented' and depend on effective bone ingrowth for fixation, these findings are of potential clinical importance.

## Competing interests

The authors declare that they have no competing interests.

## Authors' contributions

GNS conceived this study, carried out all experiment work and drafted the manuscript. PMW participated in the co-ordination of the study and the drafting of the manuscript. KJM participated in the design and coordination of this study, and participated in drafting the manuscript. All authors have read and approved the final manuscript.

## Pre-publication history

The pre-publication history for this paper can be accessed here:

http://www.biomedcentral.com/1471-2474/12/247/prepub
